# Evaluation of a smartphone-based rapid fluorescent diagnostic system for H9N2 virus in specific-pathogen-free chickens

**DOI:** 10.1007/s00705-016-2922-8

**Published:** 2016-06-10

**Authors:** Seon-Ju Yeo, Bui Thi Cuc, Haan Woo Sung, Hyun Park

**Affiliations:** Department of Infection Biology, Zoonosis Research Center, School of Medicine, Wonkwang University, Iksan, 570-749 Republic of Korea; College of Veterinary Medicine, Kangwon National University, Chuncheon, 200-701 Republic of Korea

## Abstract

Repeated interspecies transmission of H9N2 virus from poultry to humans and human infections transmitted via aerosols highlight the need for a highly sensitive, rapid diagnostic system for the detection of this virus. However, no such test exhibiting high performance has been developed. In this study, the performance of a smartphone-based rapid fluorescent diagnostic system (SRFDS) was optimized for the diagnosis of an H9N2-virus-infected animal. To suppress the nonspecific reactivity of the bioconjugate in oropharyngeal (OP) and cloacal (CL) samples derived from chickens, different blocking reagents were tested, and a mixture of casein and sucrose was found to be optimal. To assess the performance of SRFDS, OP and CL samples were obtained from specific-pathogen-free chickens and used for comparison of this method with real-time reverse transcription PCR (rRT-PCR) at time points of three, five, and seven days postinfection (dpi). The limit of detection of SRFDS was found to be 7.5 PFU/mL, which was 138-fold higher than that of a conventional colloidal-gold-based avian influenza rapid diagnostic test. In the animal study, the presence of viral antigen was monitored with SRFDS, and the relative sensitivity (relative to rRT-PCR results) was 94.44 % (17/18) and 95.23 % (20/21) in OP and CL specimens, respectively. The specificity of SRFDS was 100 %. These results imply that the diagnostic performance of SRFDS might be comparable to that of rRT-PCR for diagnosis of H9N2 in chickens and that this test can be used as a highly sensitive rapid diagnostic method in field studies on broiler poultry and wild birds.

**Electronic supplementary material:**

The online version of this article (doi:10.1007/s00705-016-2922-8) contains supplementary material, which is available to authorized users.

## Introduction

Although H9N2 avian influenza viruses generally cause only mild to moderate disease, in co-infections with other viruses and bacteria, approximately 70 % morbidity and 30 % mortality have been reported in poultry [1, 2]. In contrast to most avian influenza viruses that have a preference for alpha 2-3-linked sialic acid (SA) receptors, some H9N2 viruses are able to recognize alpha 2-6-linked SA receptors for direct transmission to humans [3]. This raises the fear that they may become pandemic through repeated interspecies transmission from poultry to humans. Moreover, as aerosol transmission of H9N2 infection has been reported, timely surveillance of H9N2 is essential [4].

To improve surveillance, an efficient and accurate rapid diagnostic method to detect H9N2 viruses in both poultry and humans is indispensable for pandemic preparedness. Studies on influenza virus shedding are important to understand the epidemiology of the virus, and they also form the basis for rational diagnostic strategies [5]. An animal model of influenza has been used to understand viral and host factors that contribute to transmission outcomes, but so far, few trials have been performed to improve rapid diagnostic tests (RDTs). As animal models are living specimens, the relationship between the amounts of viral RNA and viral antigen is biologically relevant and thus can validate the quality and accuracy of a rapid diagnostic system.

Currently, rapid diagnostic tests (RDTs) vary in their sensitivity and specificity when compared to RT-PCR. According to CDC guidelines, upper respiratory samples should be used for influenza virus RDT. In addition, the use of RDTs in hospitalized patients is not encouraged where RT-PCR is available, because the sensitivity is approximately 50–70 %, and the specificity is approximately 90–95 % [6].

To improve the accuracy and sensitivity of RDTs, many recent trials have employed fluorescent technology within this platform [7–9]. Previously, we developed a smartphone-based rapid fluorescent diagnostic system (SRFDS) with fluorescent coumarin-derived dendrimer-based bioconjugation and light-emitting diode (LED) modules to detect H5N1 virus in human throat samples [9]. However, the performance of SRFDS in the diagnosis of poultry was unclear.

Various specimens such as respiratory tract specimens and fecal specimens need to be tested using a high-performance RDT, because after the primary respiratory infection, H9N2 virus multiplies in the intestinal tract of chickens and is transmitted through feces [10, 11]. In humans, detection of influenza virus RNA and viable influenza virus in stool suggests that influenza virus can be localized to the gastrointestinal tract of children, and this could serve as a mode of transmission during seasonal and epidemic influenza outbreaks [12]. In severe cases, stool specimens have been subjected to rRT-PCR and virus isolation targeting the influenza RNA matrix (M) gene [13]. Therefore, a highly sensitive rapid diagnostic system for fecal samples is essential for efficient identification and management of influenza cases in poultry and humans.

In the current study, we assessed the capacity of the SRFDS to detect H9N2 antigen from oropharyngeal (OP) and cloacal (CL) specimens, using an animal model.

## Materials and methods

### Virus stock and titration

The H9N2 virus isolate (A/chicken/Korea/KNUSWR09/2009 (H9N2)) was derived from a broiler chicken at a traditional market in Pochun, South Korea. Virus stocks were prepared and plaque assays were performed as previously described [9].

### Real-time RT-PCR

To determine the limit of detection (LOD) of the cycle threshold (Ct) value of SRFDS, a freshly prepared virus dilution was mixed with a non-infected chicken fecal suspension (10 % w/v) [14]. Because the SRFDS uses 75 μL of sample, the same amount of virus was subjected to RNA extraction using an RNeasy Mini Kit (QIAGEN, Hilden, Germany) according to the manufacturer’s instructions.

For the animal study, 75 µL of OP and CL swab samples from chickens were used for RNA extraction. The primers and probes used to detect influenza A matrix (M) gene RNA were described previously [15]. All primers and probes were synthesized by Cosmo Genetech, South Korea.

RT-PCR was performed using a Quantitect Probe RT-PCR Kit (QIAGEN, Hilden, Germany) to determine the Ct values using a CFX96 Real-Time PCR Detection System (Bio-Rad, Hercules, CA).

### Avian influenza virus rapid diagnostic test (AIV RDT)

To evaluate the performance of SRFDS, the LOD was compared with that of a commercial avian influenza virus rapid detection test (AIV RDT) (Bionote, Hwasung, South Korea). Samples were applied following the manufacturer’s instructions. Briefly, 100 μL of the serially diluted H9N2 virus in distilled water (DW) or non-infected chicken fecal suspension (10 % w/v) was tested using the RDT, and results were read at 30 min.

### Optimization of SRFDS bioconjugate for chicken samples

Bioconjugation were performed as described previously [9]. To optimize the biocojugate for cloacal samples, different blocking agents were added during the blocking step. Briefly, 10 μL of aliphatic amine latex beads (20 nm diameter; 2 % w/v) (Life Technologies, Carlsbad, USA) were washed with phosphate-buffered saline (PBS; pH 7.5) and 100 μL of coumarin-derived dendrimer (1 mg/mL in dimethyl sulfoxide) was dispersed with the amine latex in 1 mL sodium bicarbonate buffer (0.1 M; pH 8.5). After 1 h, 0.5 mL of glutaraldehyde (8 % v/v) was additionally mixed with the complex of latex beads, and incubated for 30 min. After washing the latex beads twice with PBS, coumarin-derived dendrimer-conjugated latex beads were resuspended in 50 µL of 1 mg/mL anti-influenza nucleoprotein (NP). After vortexing, the conjugate mixture was incubated at 4 °C for 2 h. After centrifugation at 27,237 × *g* for 5 min, the collected bioconjugates were blocked for 30 min in different blocking buffers (0.1 % bovine serum albumin [BSA], 0.1 % gelatin, 0.1 % sucrose, 0.1 % casein, and mixture of 0.1 % casein and 0.1 % sucrose) and resuspended in 1 mL of storage buffer (0.1 % w/v BSA in PBS, pH 7.6) and kept at 4 °C.

### Optimization of SRFDS using a swab samples

To operate the SRFDS using a swab sample, a swab was first placed in 500 µL of lysis buffer (25 mM HEPES, 200 mM NaCl, 50 mM MgCl_2_, and 0.1 % v/v NP-40; pH 7.5), and swirled at least 10 times and left still for 10 s.

To operate the diagnostic test, 10 μL of bioconjugates were applied to the conjugate pad. After covering the strip with the strip cover, 75 mL of sample, followed by 50 μL of lysis buffer, was introduced into the predefined hole in the strip cover. The strip was kept in the dark for 15 min and fluorescent intensities were measured using the smartphone detector.

### Study group

Four-week-old SPF chickens (Namduck SPF, Sungnam, Korea) were inoculated nasally with 10^3.8^ times the 50 % egg infectious dose (EID_50_) and maintained in an SPF isolator (Biobase, Shandong, China). OP and CL swab samples from each chicken were collected at 3 (*n* = 9), 5 (*n* = 9), and 7 (*n* = 9) days postinfection (dpi). All specimens were subjected to rRT-PCR, SRFDS, and RDT. The animal experiments were approved by the Institutional Animal Care and Use Committee of Kangwon National University (KW-150414-1).

### Statistics

Means and standard deviations (SD) were calculated, and Student’s t-test and linear regression were conducted using GraphPad Prism5.0. Kappa and chi-squared tests were performed, and 95 % confidence intervals (CI) were calculated using MedCalc statistical software to compare the performance indicators.

## Results

### Procedure for SRFDS using chicken samples

**Fig. 1:**
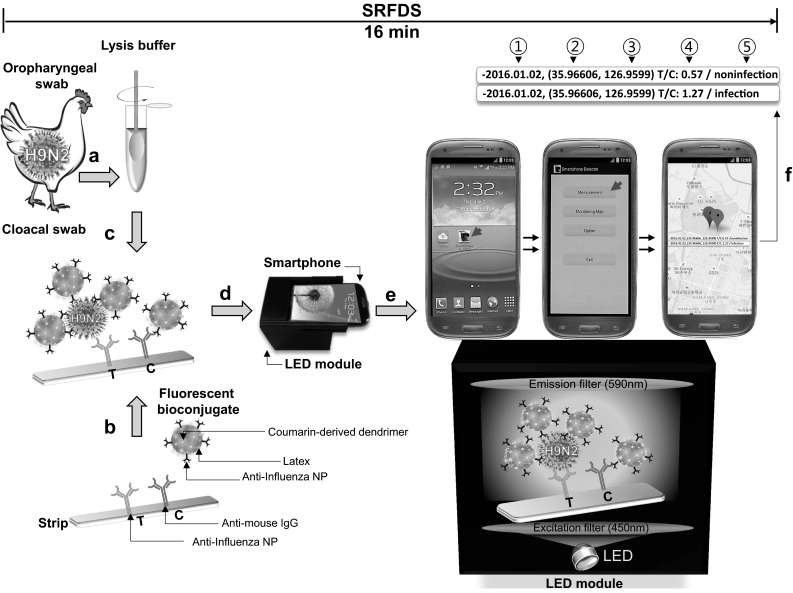
Schematic diagram of smartphone-based rapid fluorescence diagnostic system (SRFDS). (**a)** A swab from a chicken was pretreated with lysis buffer for 10 s. (**b**) A fluorescent bioconjugate was dropped onto the conjugate pad on the strip. (**c)** Subsequently, the specimen was applied to the sample pad, and sample buffer was then applied to the sample pad. **(d)** After 15 min, the smartphone camera was used to detect the fluorescent light on the strip through the LED module for 10 s. **(e)** In the LED module, the excitation light was filtered by the emission filter. To measure the fluorescence intensity, a ‘Smartphone detector’ icon (red arrow) was clicked on the screen and, subsequently, a ‘Measurement’ icon (red arrow) was clicked. **(f)** The touchscreen immediately displayed the results of the ratio of the test line (T) and control line (C) values, shown on a Google map with binary diagnostic decision as well as test date and location. Finally, all procedures were conducted within 16 min. A red balloon indicates a positive diagnostic binary decision and a green balloon indicates a negative result. 1, date of testing; 2, longitude of testing location; 3, latitude of testing location; 4, ratio between fluorescence of T and C; 5, binary diagnostic decision

First, OP and CL swabs were pretreated with 500 µL of lysis buffer for 10 s (Fig. 1a). Before application of the pretreated lysate to the strip, 10 µL of bioconjugate (latex conjugated with coumarin-derived dendrimer and antibody) was added to the conjugate pad of the diagnostic strip (Fig. 1b). Next, 75 µL of lysate was applied dropwise to the sample pad, and 50 µL of sample buffer was added to complete the lateral flow reaction (Fig. 1a–c). The details of the smartphone-based instrument can be found in our previous report [9]. After 15 min (to complete the lateral flow assay reaction), a smartphone camera was used to detect fluorescence on the strip (Fig. 1d and f). T is the fluorescence intensity of the test line, and C is the fluorescence intensity of the control line. The values of T/C of the SRFDS were used for a binary diagnostic decision depending on the cutoff value.

### Limit of detection (LOD)

**Fig. 2:**
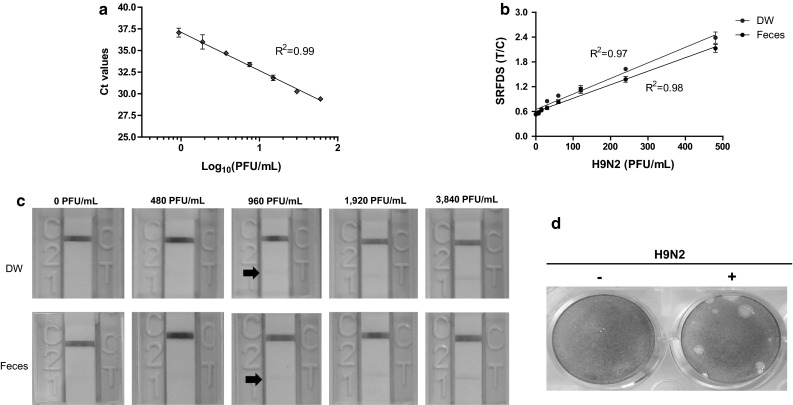
Comparison of virus detection by rRT-PCR, SRFDS, and RDT. **(a)** After preparing twofold dilutions from of samples ranging from 0.94 PFU/mL to 60 PFU/mL in 10 % (w/v) non-infected chicken fecal suspension, 75 μL of sample was subjected to RNA extraction. The eluted RNAs were used for rRT-PCR. The linear relationship between the threshold cycle (Ct) and log_10_ concentration of PFU/mL titers after regression analysis are shown. **(b)** The same amounts of virus (from 0.94 PFU/mL to 60 PFU/mL in DW and fecal suspensions) were tested using the SRFDS for 16 min. **(c)** A total of 100 μL of virus from 480 PFU/mL to 3,840 PFU/mL in DW and fecal suspensions were tested by AIV RDT for 30 min and read by the naked eye. A faint positive band in the test line (T) was detected at 960 PFU/mL (black arrow), indicating the limit of detection of AIV RDT. **(d)** The titer of the H9N2 virus was determined by plaque assay. +, infection; –, negative control

The lowest detectable virus titers for rRT-PCR and SRFDS was determined by the limit of blank (LOB) and LOD, as described previously [16]. The linear range of rRT-PCR was from 60 PFU/mL to 0.94 PFU/mL (*R*^*2*^ = 0.99). The LOB was a Ct value of 38.13, and the LOD was a Ct value of 36.34. The details of the rRT-PCR results can be found in the Supplementary Figure (Fig. S1). These values are in agreement with a previous report describing Ct values of the M gene [17]. The lowest detectable virus titer for SRFDS was also determined by the LOB and LOD as described previously [16]. In our study, the LOD of the rRT-PCR corresponded to a virus titer of 1.8 PFU/mL (Fig. 2a).

The T/C ratios of the SRFDS showed a linear range of virus detection between 0.94 × 10^0^ and 4.8 × 10^2^ PFU/mL in DW (R^2^ = 0.97) and fecal suspensions (R^2^ = 0.98) (Fig. 2b). The LOB of SRFDS was 0.53 (DW) and 0.54 (feces suspension). The LOD of the SRFDS was determined to be 0.58 (DW) and 0.56 (feces), which corresponded to 7.5 PFU/mL for H9N2 virus. In the absence of lysis buffer, the intensity of the fluorescent signal was lower and migration on the strip was less efficient than that with lysis buffer (data not shown).

In the AIV RDT, the LOD was found to be 9.6 × 10^2^ PFU/mL using both solvents, indicating that the SRFDS increased virus detection capacity 138-fold relative to AIV RDT (Fig. 2c). H9N2 plaques are shown in Fig. 2d.

When virus was dissolved in 50 % w/v feces paste and a swab was applied to SRFDS, LOD was 60 PFU/mL, which was still better than that of AIV RDT (see Supplementary Fig. S2).

### Optimization of SRFDS bioconjugate for chicken samples

**Fig. 3:**
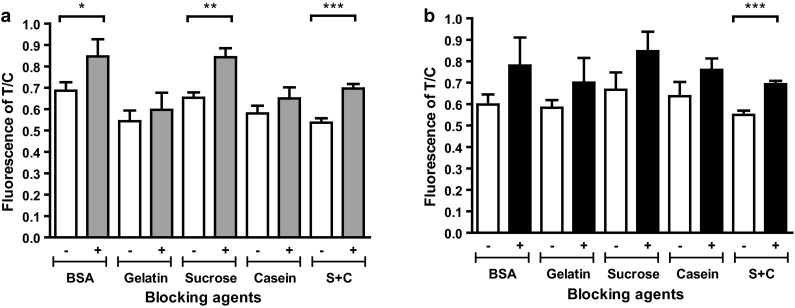
Optimization of the bioconjugate. To reduce nonspecific reactions of the bioconjugate in fecal samples, 0.1 % BSA, gelatin, sucrose, or casein was used as a blocking agent to generate the bioconjugate. They were tested against 30 PFU of H9N2 virus per mL in normal chicken oropharyngeal (OP) samples **(a)** and 10 % (w/v) non-infected chicken fecal suspension (**b**). Data (*n* = 3) are shown as mean ± SD, Student’s *t*-test. +, H9N2 virus; –, negative control; S + C, mixture of 0.1 % sucrose and 0.1 % casein (*, *P* < 0.05; **, *P* < 0.01; ***, *P* < 0.0001)

Blocking solutions based on BSA, gelatin, dry milk, or casein are known to prevent nonspecific reactions by blocking hydrophobic interaction between proteins and ionic or electrostatic interactions [18]. To optimize the function of SRFDS for OP and CL samples through the suppression of nonspecific reactions during diagnostic testing, several blocking agents were added during the blocking step of the bioconjugate. After washing away the unbound blocking reagent, all bioconjugates were stored in the same buffer, 0.1 % BSA in PBS. The different bioconjugates were evaluated with both OP and CL samples in a diagnostic strip test (Fig. 3). In our study, a sucrose-treated bioconjugate was the most efficient for OP specimens (Fig. 3a); however, it was not effective in suppressing the nonspecific reaction in the CL specimen. The addition of 0.1 % casein to 0.1 % sucrose efficiently suppressed the nonspecific reaction in the fecal sample (Fig. 3b). Therefore, a mixture of 0.1 % sucrose and 0.1 % casein was used for further screening of OP and CL samples from chickens.

### Analysis of the diagnostic performance of SRFDS using chicken samples

OP and CL samples from individual chickens infected with H9N2 virus were tested in parallel by rRT-PCR, SRFDS, and AIV RDT.

**Fig. 4:**
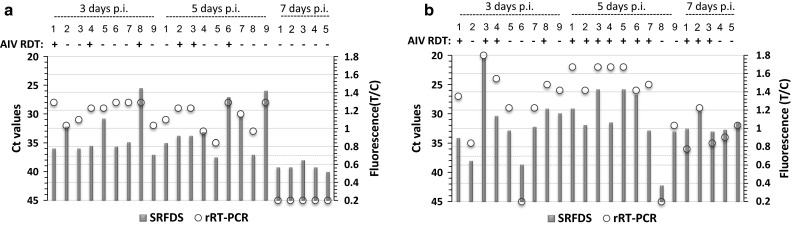
Comparison of Ct values with T/C of SRFDS and AIV RDT. SPF chickens were inoculated nasally with 10^3.8^ EID_50_ of H9N2 virus. Oropharyngeal swab (**a**) and cloacal swab (**b**) samples were collected at 3, 5, and 7 days post-inoculation (dpi). All swabs from each chicken were analyzed using the three assays. For antigen detection, AIV RDT and SRFDS were tested with OP and CL samples. RNA levels were confirmed by rRT-PCR. A red circle in the graph indicates the Ct value of the rRT-PCR. A green bar indicates the T/C ratio measured by SRFDS. A dashed line indicates the cutoff value of T/C using SRFDS. p.i., postinfection; +, AIV RDT positive; –, AIV RDT negative

For rRT-PCR analysis targeting the M gene, samples with Ct values ≤36 were considered positive; positive Ct values were observed up to 5 dpi. At 7 dpi, Ct values of all OP samples were not in the positive range (Fig. 4a). In contrast, only CL samples continued to yield positive Ct values (≤36) up to 7 dpi (5/5), implying that H9N2 virus was not actively multiplying in the oropharynx at 7 dpi in our experiment (Fig. 4b).

**Fig. 5:**
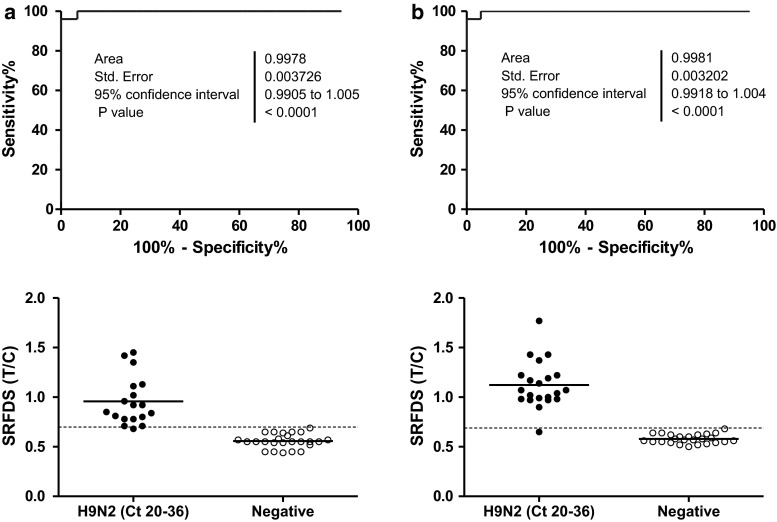
Receiver operating characteristic (ROC) analysis. ROC curve analysis was conducted to evaluate the diagnostic performance of SRFDS with OP specimens (**a**) and CL samples (**b**) with Ct values of 20-36. The cutoff value (dashed line) of T/C was used to determine if each T/C value indicated a positive or negative result (bottom panel of a and b) in the animal study, determining sensitivity and specificity. H9N2 (Ct 20-36), specimen with Ct values of 20-36; Negative, non-infected normal and rRT-PCR-negative specimens in both OP and CL samples

To compare the clinical diagnostic performance of SRFDS to that of rRT-PCR, the cutoff value for T/C was determined from a receiver-operating characteristic (ROC) curve analysis using OP (Fig. 5a upper panel) and CL samples (Fig. 5b upper panel). The cutoff value of T/C was 0.69 (positive if T/C values ≥0.69, negative otherwise). For the SRFDS, OP samples yielded positive T/C values up to 5 dpi, with one false negative result, and all samples were negative at 7 dpi. Therefore, for OP samples, SRFDS showed 100 % (9/9) (3 dpi), 88.9 % (8/9) (5 dpi), and 0 % (0/5) (7 dpi) sensitivity. At 7 dpi, the OP and CP of four chickens were all negative, and thus they were included in the negative chicken group.

In CL samples, positive T/C values were seen up to 7 dpi, with one false negative result at 3 dpi. Out of five infected individual chickens, five had positive T/C values at 7 dpi, indicating that for CL samples, SRFDS had 88.9 % (8/9) (3 dpi), 100 % (9/9) (5 dpi), and 100 % (5/5) (7 dpi) sensitivity. The AIV RDT screening test showed lower sensitivity than that of the SRFDS test for both specimens.

As seen in Table 1, the sensitivity of SRFDS was comparable to that of rRT-PCR in specimens with Ct values of 20-30; however, AIV RDT demonstrated only 50-80 % sensitivity in both types with Ct values ≤30. While AIV RDT performance was lower than 50 % in specimens with Ct values of 31-36, SRFDS showed more than 80 % sensitivity in these samples.

**Table 1:** Comparison of rRT-PCR with SRFDS and RDT using clinical samples from experimentally infected SPF chickens

Specimen^a^	Sensitivity (%)						Specificity (%)	
	Ct 20–30		Ct 31–36		Ct 20–36		RDT	SRFDS
	RDT	SRFDS	RDT	SRFDS	RDT	SRFDS		
OP	54.55 (6/11)	100 (11/11)	0 (0/7)	85.70 (6/7)	33.33 (6/18)	94.44 (17/18)	100 (25/25)	100 (25/25)
CL	80.00 (12/15)	100 (15/15)	50.00 (3/6)	83.33 (5/6)	71.42 (15/21)	95.23 (20/21)	100 (25/25)	100 (25/25)

^a^Chickens were inoculated nasally with 10^3.8^ EID_50_ of H9N2. Oropharyngeal (OP) and cloacal (CL) samples of individual chickens were collected at 3, 5, and 7 days post-inoculation

In OP specimens with Ct values of 20-36, the sensitivity of SRFDS and AIV RDT was 94.44 % (17/18) (Kappa; 0.956, 95 % CI; 0.871-1.000, *P* < 0.0001) and 33.33 % (6/18) (Kappa; 0.385, 95 % CI; 0.147-0.622, *P* < 0.001), respectively. In CL samples with Ct values of 20-36, the sensitivity of the SRFDS and AIV RDT was 95.23 % (20/21) (Kappa; 0.959, 95 % CI; 0.879-1.000, *P* < 0.0001) and 71.42 % (15/21) (Kappa; 0.738, 95 % CI; 0.548-0.927, *P* < 0.0001), respectively.

The rRT-PCR-negative OP (*n* = 25) and CL samples (*n* = 25) were all negative by the SRFDS and AIV RDT.

Therefore, the results of the AIV RDT showed fair agreement with those of the rRT-PCR, and the results of the SRFDS showed very good agreement with those of the rRT-PCR for both types of specimens.

## Discussion

When an avian influenza outbreak occurs, primary screening tests at the clinical level are used to detect the presence of these viruses [19]. Recently, the ability of H9N2 to cause respiratory infection was reported to be about 40 times greater than its ability to cause gastrointestinal infection, emphasizing that urgent attention is needed to stop the airborne transmission of influenza virus [10].

Antigen detection systems can reflect the infectious period of the influenza virus (H1N1) in a ferret model. Herein, a shift in antigen-detection test results from positive to negative coincided with a rapid decrease in viable virus titer, and cessation of transmission occurred at the point at which the Ct value was approximately 35 in rRT-PCR [20]. This corresponds to the proposed SRFDS performance, which shows positive antigen detection up to a Ct value of 36 in our study.

Typically, H9N2 virus replicates predominantly in the respiratory tract of chickens, but it is also occasionally isolated from the cloacal swab [11]. Our results of SRFDS and rRT-PCR showed the possibility of the presence of a larger amount of H9N2 virus in cloaca samples than in upper respiratory samples. There is one study in which the detection rate was higher in feces than in the trachea [21].

By studying the virus antigen profile determined by SRFDS in both OP and CL samples, we found sensitivities of 94.44 % and 95.23 % in OP and CL samples, respectively. However, for individual chickens, this sensitivity was improved to 100 %, as the results of the OP and CL samples complemented each other. This observation suggests that the combined results of both OP and CL samples can significantly increase the diagnostic performance of a rapid diagnostic system and result in a better diagnostic binary decision using SRFDS when compared to using only separate diagnostic results (derived from different specimens). Therefore, we might need to reconsider typical RDT platforms for efficient field testing to enable simultaneous testing of both OP and CL specimens, which confers high sensitivity comparable to that of rRT-PCR.

To confirm the performance of SRFDS, feces derived from broiler chickens and wild birds collected in the environment should be subjected to further optimization.

In conclusion, the optimization of SRFDS has improved its clinical diagnostic performance compared to conventional AIV RDT. We believe that the SRFDS can be used in poultry surveillance and human AIV case management.

## Electronic supplementary material

Below is the link to the electronic supplementary material.

Supplementary material 1 (DOCX 187 kb)
