# Supplementary material for: Evaluation of a smartphone-based rapid fluorescent diagnostic system for H9N2 virus in specific-pathogen-free chickens
Source: Arch Virol. 2016 Jun 10;161:2249–56. doi: 10.1007/s00705-016-2922-8 (PMC4947103; doi:10.1007/s00705-016-2922-8)
Supplement: Supplementary file 1 — Supplementary material 1 (DOCX 187 kb) [file 705_2016_2922_MOESM1_ESM.docx]

**Supplementary Information**

**Article title**

**Evaluation of smartphone-based rapid fluorescent diagnostic system for H9N2 virus in specific pathogen free chicken**

**Article author**

Seon-Ju Yeo^‡1^, Bui Thi Cuc^‡1^, Haan Woo Sung^‡2^, Hyun Park^§1^

**Supplementary Figures**

**Figure S1.** **Ct values of M gene by rRT-PCR**

**Figure S2. Efficiency of SRFDS for fecal pastes**

**Supplementary Figure**


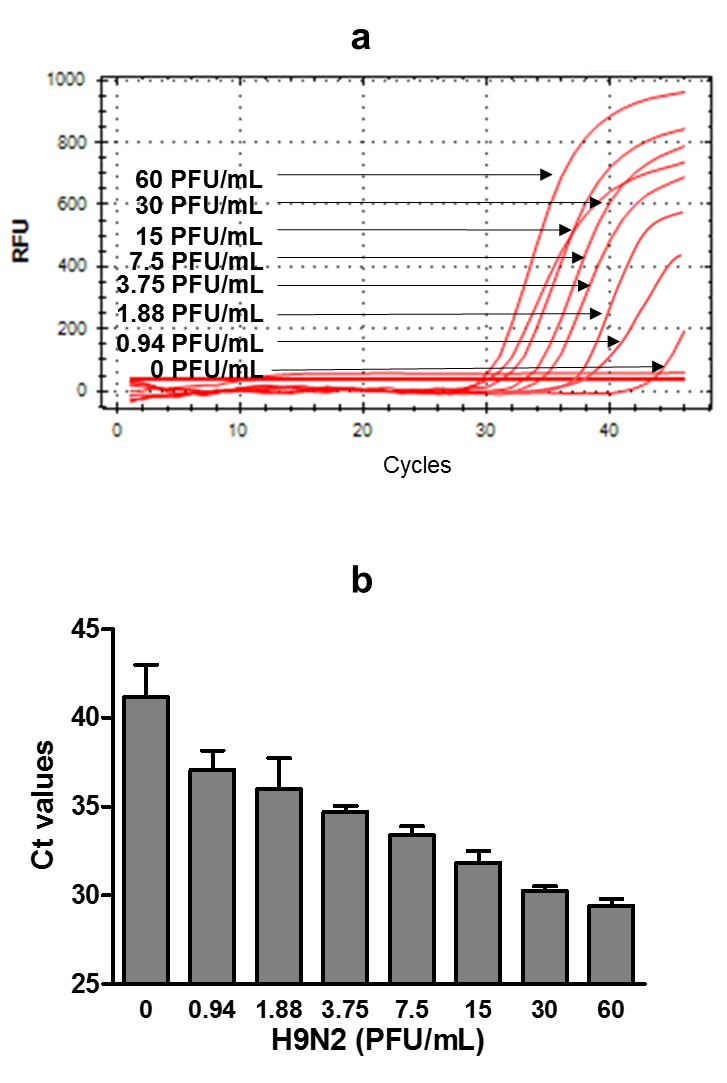


**Figure S1. Ct values of M gene by rRT-PCR.** After preparing two-fold dilutions from 0.94 PFU/mL to 60 PFU/mL in 10% (w/v) non-infected chicken fecal suspension, 75 μL of samples was subjected to RNA extraction. The eluted RNAs were employed for rRT-PCR assay (a). The mean of Ct values (*n* = 3) were plotted on the y-axis and amount of input virus titer on the x-axis (b).

**Figure S2. Efficiency of SRFDS for fecal pastes.** To measure the efficiency of SRFDS for 50% w/v feces, the feces paste including H9N2 virus were generated. Serially diluted H9N2 virus (400 mL) were swirled in 0.4g of non-infected chicken feces to make a paste of feces (50 % w/v). A swab derived from each feces pastes were dissolved in 500 μL of lysis buffer for 10 seconds and then 75 μL of samples were tested in SRFDS. LOD was calculated as 60 PFU/mL, which was still better than that of AIV RDT.
